# Leptin Gene Protects Against Cold Stress in Antarctic Toothfish

**DOI:** 10.3389/fphys.2021.740806

**Published:** 2021-12-15

**Authors:** Ying Wang, Huamin Wang, Linghong Hu, Liangbiao Chen

**Affiliations:** ^1^International Research Center for Marine Biosciences, Ministry of Science and Technology, Shanghai Ocean University, Shanghai, China; ^2^Key Laboratory of Exploration and Utilization of Aquatic Genetic Resources, Ministry of Education, Shanghai Ocean University, Shanghai, China; ^3^Shanghai Collaborative Innovation for Aquatic Animal Genetics and Breeding, Shanghai Ocean University, Shanghai, China

**Keywords:** *leptin-a*, positive selection, polar fish, STAT3 signaling, p53, *Dissostichus mawsoni*

## Abstract

Leptin is a cytokine-like peptide, predominantly biosynthesized in adipose tissue, which plays an important role in regulating food intake, energy balance and reproduction in mammals. However, how it may have been modified to enable life in the chronic cold is unclear. Here, we identified a *leptin-a* gene (*lepa*) in the cold-adapted and neutrally buoyant Antarctic toothfish *Dissostichus mawsoni* that encodes a polypeptide carrying four α-helices and two cysteine residues forming in-chain disulfide bonds, structures shared by most vertebrate leptins. Quantitative RT-PCR confirmed that mRNA levels of the *leptin-a* gene of *D. mawsoni* (DM-*lepa*) were highest in muscle, followed by kidney and liver; detection levels were low in the gill, brain, intestine, and ovary tissues. Compared with *leptin-a* genes of fishes living in warmer waters, DM-*lepa* underwent rapid evolution and was subjected to positive selection. Over-expression of DM-*lepa* in the zebrafish cell line ZFL resulted in signal accumulation in the cytoplasm and significantly increased cell proliferation both at the normal culture temperature and under cold treatment. DM-*lepa* over-expression also reduced apoptosis under low-temperature stress and activated the STAT3 signaling pathway, in turn upregulating the anti-apoptotic proteins bcl2l1, bcl2a, myca and mdm2 while downregulating the pro-apoptotic baxa, p53 and caspase-3. These results demonstrate that DM-*lepa*, through STAT3 signaling, plays a protective role in cold stress by preventing apoptotic damage. Our study reveals a new role of *lepa* in polar fish.

## Introduction

The obesity gene was first discovered on chromosome 6 in morbidly obese mice in 1994; the gene contains a 501-bp open reading frame (ORF) which encodes a polypeptide called leptin (Zhang et al., 1994). The leptin gene has subsequently proven to be highly conserved in other mammals, with similarities of up to 96% between rats and mice and 83% among rats, mice, and humans (Murakami and Shima, 1995; Sasaki et al., 1996; Konfortov et al., 1999). Studies on fish leptin were initiated more recently. In 2005, with reference to human and murine leptin, a leptin homolog was cloned in the Japanese pufferfish (*Takifugu rubripes*) *via* comparative genomics, promoting studies on ichthyic leptin (Kurokawa et al., 2005). However, the sequence similarity among bony fishes is relatively low. Further, fishes harbor multiple leptin homologs owing to gene duplication. For example, Japanese medaka (*Oryzias latipes*) harbors *lep-a* and *lep-b*, which are located on chromosomes 6 and 23, respectively, (Kurokawa and Murashita, 2009). Sequence alignment has revealed that the sequence similarity between *lep-a* and *lep-b* in Japanese medaka is only 16.5%. Similarly, zebrafish (*Danio rerio*) also have two homologous genes, *lepa* and *lepb* (Gorissen et al., 2009), and the amino acid sequence similarity between the two is also low at 24%. The *T. rubripes*, *Salvelinus alpinus* (Arctic char), and *Pelteobagrus fulvidraco* genomes carry the *leptin-a* gene but not *leptin-b* (González et al., 2000; Kurokawa et al., 2005; Frøiland et al., 2010; Choi et al., 2014). In contrast, in *Cyprinus carpio* (common carp) and *Salmo salar* (Atlantic salmon), *leptin-a* is duplicated (Huising et al., 2006; Rønnestad et al., 2010). This suggests that the functions of leptins in fish may be more complicated than in mammals.

Recent studies indicate that leptin genes undergo rapid evolution and are subject to positive selection in many animal clades, including seals (Hammond et al., 2005; Yu et al., 2011), cetaceans (Yu et al., 2011), pikas (*Ochotona* spp.; Yang et al., 2008, 2011), heterothermic bats (Yuan et al., 2011), and primates (Benner et al., 2002; Siltberg and Liberles, 2002; Gaucher et al., 2003; Berglund et al., 2005; Yang et al., 2011). These findings suggest that selection on leptin may be an important contribution to environmental adaptation. However, the question of whether the leptin gene of fishes living in extreme cold environments has also undergone evolutionary selection is of interests, but has been not reported.

*Dissostichus mawsoni*, belonging to the Antarctic notothenioids, has adapted to the extremely low temperature of the ocean surrounding Antarctica. It has extensive lipid (mostly triglyceride) deposits under the skin and in the musculature (Chen et al., 2019), compared to other fish species in the same depth of water. As leptin plays a central role in the regulation of lipid metabolism, we speculate that the leptin of *D. mawsoni* has undergone adaptive functional evolution, in addition to regulating fat deposition, it also plays an important role in cold temperature adaptation. Recent studies have shown that leptin can inhibit cell apoptosis by activating the STAT3 signaling pathway, leading to increased expression of anti-apoptotic genes and a decrease of pro-apoptotic gene expression (McGaffin et al., 2009). Furthermore, it has been shown in many cell types that STAT3 activation is associated with cell survival and proliferation (Kanda et al., 2004; Yu et al., 2014; Banerjee and Resat, 2016). There is also evidence that leptin exerts its anti-apoptotic effect by reducing the level of p53 (Toro et al., 2014). In this study, a *leptin-a* gene (*lepa*) from *D. mawsoni* was cloned and transfected to a fish-liver cell line. We revealed that *D. mawsoni* leptin-a provides cells with potent protection from the stresses of low-temperatures.

## Materials and Methods

### Cloning and Tissue Expression Analysis of the *leptin-a* Gene of *D. mawsoni*

The fish specimens used in the present study were the same as those used in our previous study (Chen et al., 2019). Total RNA was extracted from the liver, using TRIzol (Invitrogen, Carlsbad, CA, United States), tested for purity *via* agarose gel electrophoresis, and spectrophotometrically quantified (NanoDrop2000, Thermo Fisher, CA, United States). One microgram of total RNA was reverse-transcribed into single-stranded cDNA, using the Prime Script RT Kit (Takara, Japan). Based on the cDNA library of *D. mawsoni* sequencing results previously obtained by our laboratory, primers *lepa*F/*lepa*R (Table 1) for cloning were designed by screening the sequence in comparison with its homologs. PCR was performed using rTaq polymerase (TaKaRa, Japan) according to the following protocol: 95°C for 5 min, 34 cycles of 95°C for 30 s, 58°C for 30 s, 72°C for 30 s. For tissue expression analysis, a FastStart Universal SYBR Green Master Kit (Roche, Germany) and LightCycler 480 (Roche, Germany) were used for qPCR with 18S as the reference gene (Lauth et al., 2002). The 18S primer was as used in the previous study. Thermal cycling was performed using an initial denaturation step of 95°C for 3 min followed by 40 cycles of 95°C for 10 s, annealing temperature for 20 s, and 72°C for 20 s. The relative tissue expression levels of *lepa* were calculated by the 2^–ΔΔCt^ method. The primer sequences are shown in Table 1.

**TABLE 1:** Primers for *D. mawsoni* lepa cloning and tissue expression.

Primer name	Primer sequence (5′-3′)	Application
lepaF	GGGTTTGGAGAGAGGTGAG	Cloning
lepaR	TGTGGCCAGCGACTTACATA	
lepaqF	GATCGACCTTGAGACGCACT	qPCR
lepaqR	TCTTGGGATGGTTGGTCAGC	
18SqF	GTTCGATTCCGGAGAGGGAG	Reference gene
18SqR	CCTTCCTTGGATGTGGTAGCC	

### Selective Pressure Analysis

To test the selective pressure of the *lepa* gene of *D. mawsoni*, we compared its sequence with those six fish species (*L. japonicus, L. maculatus, S. chuatsi, E. coioides, O. latipes*, and *D. rerio*) from relatively warm waters (more than 10°C). Sequences were aligned using MEGA 6 (Tamura et al., 2013), which generated the phylogenetic tree for *lepa* genes *via* the maximum-likelihood method. Two likelihood-ratio tests (LRTs) were performed by means of the branch and site models of codon evolution using the *codeml* program (Yang, 2007) from the PAML package (Yang, 1997). Significance (*P* value < 0.05) of the compared LRTs was conducted by the *chi2* program from the PAML package.

### Construction of DM-*lepa* Expression Vector

For *lepa* over-expression, the CDS sequence of *lepa* was cloned into the constructed pTol2-actin-egfp plasmid (the promoter was β-actin from zebrafish) to produce pTol2-actin-lepa-egfp. Due to the lack of a specific *lepa* antibody, we also introduced the *His*-tag before the *lepa* termination codon to produce the LEPA-HIS fusion protein. In this way, we could use commercial HIS antibodies to detect LEPA protein expression for convenience. The expression plasmids then were transferred into *E. coli* DH-5α competent cells, plated on culture dish containing 100 μg⋅mL^–1^ kanamycin (Sangon, Shanghai, China) and cultured in a 37°C incubator for 12 h before analysis *via* individual bacterial colony PCR. The positive clones obtained *via* sequencing underwent expansion culturing, and plasmids were extracted using a plasmid extraction kit (Promega, Wisconsin, United States) in accordance with the manufacturer’s instructions and stored at −20°C until use.

### Culture and Plasmid Transfection of the Zebrafish Liver Cells

The commercially available zebrafish liver (ZFL) cell line (American Type Culture Collection, ATCC, VA, United States) was cultured in culture medium containing 89% DMEM/F-12, 10% FBS, and 1% penicillin/streptomycin at 28°C and 5% CO_2_ (i.e., conditions for optimum growth). Lonza Nucleofector (Lonza, Germany) was used for transfection at 70–80% confluence. G418 was added to the transfected ZFL cells after expansion culturing to select for successfully transfected cells. The concentration of G418 for the first week was 1,000 mg⋅mL^–1^ and 500 mg⋅mL^–1^ thereafter; these were the concentrations at which most of the non-transfected cells died. The G418 was then discarded, and the successfully transfected cells highlighted with green fluorescence.

### Protein Expression and Subcellular Localization of DM-*lepa* in Zebrafish Liver Cells

The screened ZFL cells were cultured under optimal growth conditions for 48–72 h and then mixed with RIPA Lysis and Extraction Buffer (Thermo, CA, United States) and 1 mmol⋅L^–1^ PMSF for protein extraction. The protein concentration was measured using a BCA Kit (Thermo, CA, United States) in accordance with the manufacturer’s instructions, and proteins (30 μg per sample) were separated *via* SDS-PAGE and electro-transferred to a polyvinylidene difluoride nitrocellulose membrane (Millipore, Germany). Antibodies used in the assays included commercial HIS primary antibody (1:1000, Abcam, United Kingdom), β-actin (1:5000, Hua An, Hangzhou, China), and goat anti-mouse secondary antibody (1:5000, Hua An, Hangzhou, China). The proteins were visualized by enhanced chemiluminescence detection reagents (Beyotime, China).

To identify the localization of these proteins, ZFL cells were cultured in 24-well microporous plates (Corning, NY, United States) and fixed using 4% paraformaldehyde at ambient temperature for 30 min. Cells were then washed twice with PBS and blocked with treating buffer (0.1% Triton X-100, 1% BSA) for 30 min. Thereafter, the cells were counterstained with 2 μg⋅mL^–1^ of 4′,6-diamidino-2-phenylindole (DAPI, Sigma, St. Louis, MO, United States) for 5 min and washed thrice with water for 5 min each time. An inverted fluorescence microscope (ZEISS, Germany) was used to examine the ZFL cells and to obtain photographs.

### Cell Viability Assays by Cell Counting Kit-8

A Cell Counting Kit-8 (CCK-8, Beyotime, China) was used to measure cell proliferation according to the manufacturer’s protocol. The over-expression *lepa* group and control group cells were inoculated into a 96-well plate (Corning, NY, United States), and 10 μL CCK-8 reagent was added to each well at the time of harvest. After that, the cells were incubated at 28°C and 18°C for 0, 24, 48, 72, and 96 h, respectively. The low treatment temperature (18°C) refers to our previous research (Chen et al., 2017). At the indicated time points, the absorbance at 450 nm was measured to determine the cell viability using the microplate reader (Synergy H4 Hybrid Reader, BioTek, United States). The data are representative of three independent experiments in triplicate.

### ROS Detection by Flow Cytometry

The generation of intracellular ROS was determined using a fluorescein-labeled dye, 2′,7′-dichlorofluorescein diacetate (DCFH-DA, Beyotime, China), following the manufacturer’s protocol. Briefly, the DCFH-DA fluorescent probe was added to cells (5 × 10^4^ cells/mL) followed by incubation for 20 min at their respective temperatures. Finally, cells were washed by PBS and then analyzed by a BD Accuri C6 flow cytometer (BD Biosciences, United States). FlowJo software (FlowJo, Ashland, OR, United States) was used to analyze the data.

### Cell Apoptosis Analysis

Hoechst/propidium iodide (PI) staining (Beyotime, China) was performed to assess the percentage of dead cells or late-stage apoptotic cells. Briefly, the over-expression *lepa* group and the control group cells were inoculated into a 24-well plate (Corning, NY, United States), then 1 mL of fresh growth medium was added to each well. Incubation of the cells proceeded at 28°C and 18°C until they reached 80–90% confluence, respectively. After that, PI (1 μg/mL) and Hoechst (1 μg/mL) was added to each well and incubation continued for 30 min at room temperature. All samples were observed and photographed with a Zeiss fluorescence microscope (Zeiss, Germany). The percentage of apoptotic cells was calculated using ImageJ software (National Institutes of Health, Bethesda, MD, United States).

### Western Blot Analysis of Signal-Transduction Proteins

Total cellular proteins of each sample were separated by SDS-PAGE electrophoresis, and western blots were conducted as our previous study described. After blocking, the membranes were overlaid with primary antibodies for cleaved caspase-3 (1:1000, Hua An, Hangzhou, China), STAT3 (1:1000, Hua An, Hangzhou, China), and p53 (1:2000, Hua An, Hangzhou, China) overnight at 4°C, then incubated with secondary antibody at 37°C for 1 h. Proteins were visualized by enhanced chemiluminescence detection reagents (Beyotime, China). The endogenous control used was β-actin.

### Analysis by qRT-PCR of Genes Involved in Signal-Transduction Pathway

Total RNA was extracted from cells using TRIzol (Invitrogen, United States) reagent according to the manufacturer’s instructions. The methods for RNA quantity, reverse transcription and real-time PCR were as mentioned above. Supplementary Table 1 presents the primer sets used. Relative expression levels were calculated using the 2^–ΔΔCT^ method, with β-actin as the reference gene for normalization.

### Sequence Analysis of the DM-*lepa* Gene

ORFfinder^1^ was used to predict the ORFs and encoded amino acid sequence of DM-*lepa*. The NCBI BLASTP tool was used to search for the homologous gene sequences of *leptin* in different species, including *Lateolabrax japonicus* (Gene Bank accession number: AHI85768.1), *Lateolabrax maculatus* (Gene Bank accession number: QFQ51510.1), *Siniperca chuatsi* (Gene Bank accession number: AHH86062.1), *Epinephelus coioides* (Gene Bank accession number: AMR58943), *O. latipes* (Gene Bank accession number: NP_001098190.2), *D. rerio* (Gene Bank accession number: NP_001122048.1), and *Homo sapiens* (Gene Bank accession number: NP_000221.1). The conserved motifs of the LEPA protein were analyzed using MEME^2^. The online tool ProtParam^3^ by ExPASy was used to predict the molecular weight of the DM-LEPA protein, its theoretical isoelectric point, instability index, aliphatic index, and grand average of hydropathicity. The online tool SignalP 4.0^4^ was used to predict signal peptide sequences. The online tool TMHMM Server 2.0^5^ was used to predict the transmembrane domain. NetPhos 2.0 Server^6^ was used to predict the Ser, Thr, and Tyr phosphorylation sites. NetNGlyc 1.0 Server^7^ was used to predict glycosylation sites. The SWISS-MODEL server^8^ was used to analyze higher-order protein structure.

### Statistical Analyses

GraphPad Prism 8 (GraphPad Software, United States) was used for statistical analyses. All experiments were performed at least in triplicate. All data were expressed as the mean ± SD. Comparisons between two groups were performed using Student’s *t*-test, and comparisons among multiple groups were performed using two-way ANOVA. *P* < 0.05 were considered statistically significant. One asterisk, two asterisks and three asterisks indicate < 0.05, *P* < 0.01 and *P* < 0.001, respectively.

## Results

### Identification and Molecular-Structure Analysis of the DM-*lepa* Gene

**FIGURE 1:**
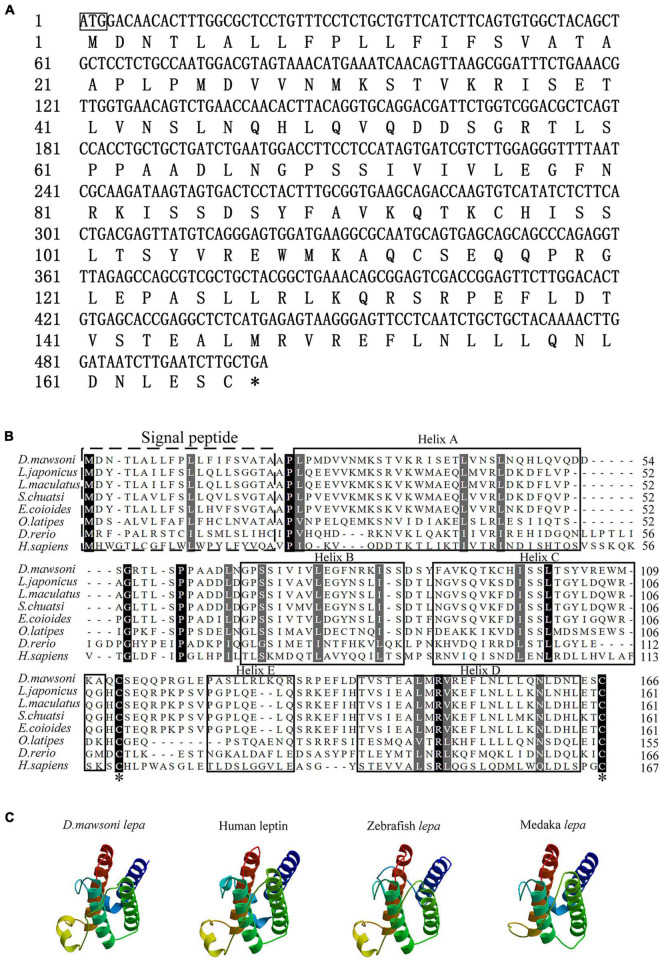
Characterization of *D. mawsoni* lepa. **(A)** Coding sequence and encoded amino acid sequence. The box and the asterisk indicates the start codon, the stop codon, respectively. **(B)** Alignment of *leptin-a* homologs among the vertebrates. The signal peptide and conserved function domains are indicated with the boxes. Identical amino acid residues are shown in black and the amino acids with >50% similarity shown in gray. Two asterisks indicate the conserved cysteine residues, which form a single disulfide bridge. **(C)** Predicted tertiary structure in different species. Four α-helices are displayed in brown, blue, dark green, and green helices.

The coding sequence of the *leptin-a* gene in *D. mawsoni* has a 501-bp ORF to encode 167 amino acid residues (Figure 1A). The initial 20 amino acid residues at its N terminus function as the signal peptide (Figure 1B). The relative molecular weight was determined to be at about 16 kDa, the theoretical isoelectric point at 6.73, the instability index at 51.50, the aliphatic index at 99.82 and the grand average of hydropathicity at −0.092, suggesting a hydrophilic protein. Multiple sequence alignments indicated that *lepa* of *D. mawsoni* shared a similarity of 71.43% with human leptin, as well as 55.42, 56.02, 57.23, 58.43, 38.55, and 18.87% with *L. japonicus*, *L. maculatus*, *S. chuatsi*, *E. coioides*, *O. latipes*, and *D. rerio*, respectively, indicating a relatively low sequence similarity (<60%) among the fish species, although two cysteine residues forming disulfide bonds were conserved in vertebrates (Figure 1B). Prediction of the 3D structure also illustrated the structural similarity among fish and higher vertebrates, all of which carried four α-helices and irregular corners to form a hollow barrel structure (Figure 1C).

### Expression Patterns of the DM-*lepa* mRNA

**FIGURE 2:**
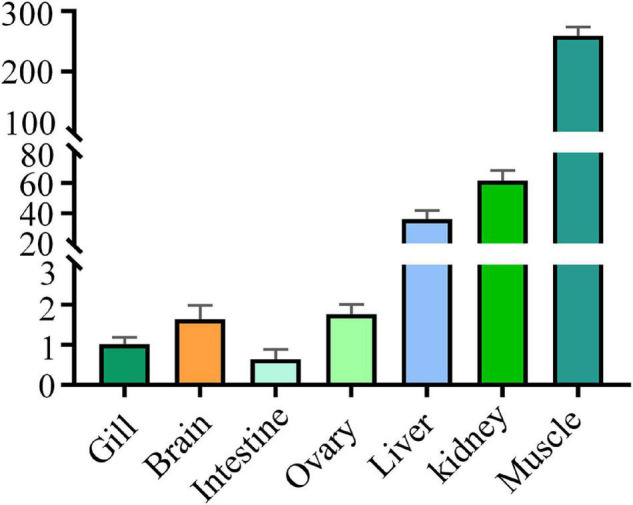
Expression patterns of DM-lepa in different tissues. The *y* axis indicates the relatively expression level with associated standard error bars, *n* = 3 (biological replicates), **P < 0.01, ***P < 0.001.

Levels of the DM-*lepa* mRNA were analyzed *via* quantitative RT-PCR, normalized relative to 18S mRNA of *D. mawsoni*. Transcription of DM-*lepa* mRNA was highest in muscle and lowest in intestine tissues. The next highest levels were observed in kidney and liver while weak expression was found in gill, brain, and ovary (Figure 2).

### Analysis of Selective Pressure

**FIGURE 3:**
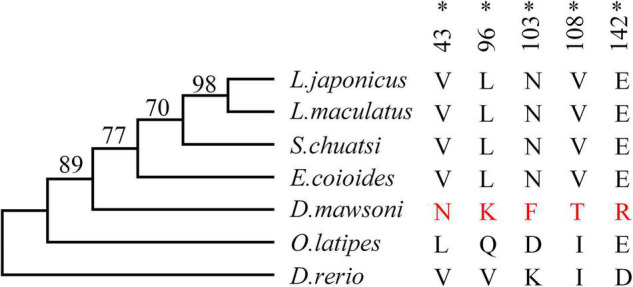
Positive selection analysis. Numbers at the branches indicate the bootstrap support. Amino acids marked in red are positive selection sites for *D. mawsoni*, *P < 0.05.

Test of positive selection for the DM-*lepa* gene was carried out using the *codeml* program in the PAML package with the maximum likelihood codon model. The phylogenetic tree was constructed by MEGA 6 using the maximum likelihood model (Figure 3). The PAML branch model calculated the LRT test statistics to be 2Δℓ = 18.359215, *p* = 0.000018, and df = 1 (Supplementary Table 2). The ω ratio for the DM-*lepa* branch (ω_1_ = 1.71507) was significantly higher than the fish species from warmer waters (ω_0_ = 0.27320). Furthermore, estimations of the PAML branch-site models identified 5 sites (43N, 96K, 103F, 108T, and 142R) under positive selection (Figure 3 and Supplementary Table 3), suggesting that DM-*lepa* underwent a rapid evolution.

### Over-Expression and Subcellular Localization of DM-*lepa* in Zebrafish Liver Cells

**FIGURE 4:**
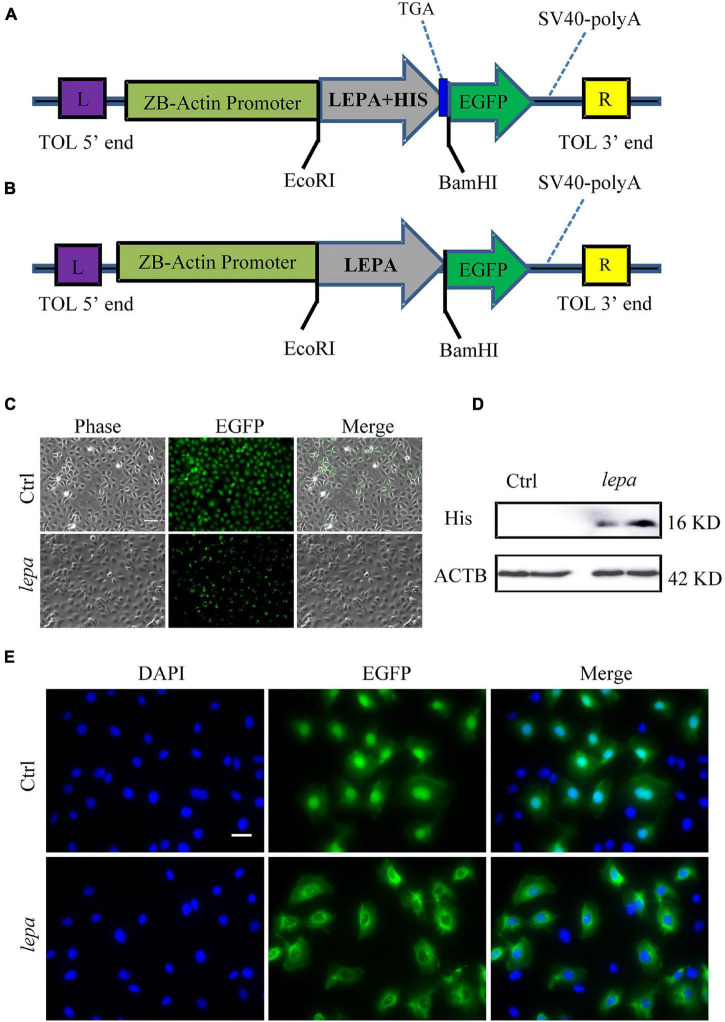
Over-expression of DM-lepa in ZFL cells. **(A,B)** DM-Lepa expression vector map. **(C)** Fluorescence observation after transfection of ZFL cells with empty plasmid and DM-lepa gene eukaryotic expression plasmid. Scale bar, 50 μm. **(D)** Western blot analysis of DM-LEPA in ZFL. **(E)** Assay of subcellular localization in ZFL cells. Green fluorescence show signal of DM-LEPA and blue nuclei stained with DAPI. Scale bar, 20 μm.

Since the *D. mawsoni* population occupies extremely cold temperatures, temperature was expected to be the most influential factor in determining evolution of DM-*lepa*. We investigated *in vitro* the functions of DM-*lepa*, because of the difficulty of artificial breeding. Owing to the lack of a commercial antibody specific to DM-LEPA, we constructed the pTol2-lepa-His-egfp expression vector (Figure 4A) to help detect the counterparts of DM-LEPA from the total ZFL cell lysates using a commercial anti-*His* tag monoclonal antibody. We eliminated the stop codon and *His* tag in the expression vector (Figure 4B) and transfected ZFL cells (Figure 4C) to identify the subcellular localization of DM-LEPA. Western-blot analysis of the total protein in ZFL cells indicated that the molecular weight of the mature protein at 16.04 kDa was consistent with predictions (Figure 4D). Detection of green fluorescent proteins indicated that the DM-LEPA protein was primarily localized in the cytoplasm rather than the nucleus (Figure 4E), a feature suggestive of a secretory protein.

### The Effect of Over-Expression of DM-*lepa* on Cell Apoptosis, Proliferation, and ROS Content

**FIGURE 5:**
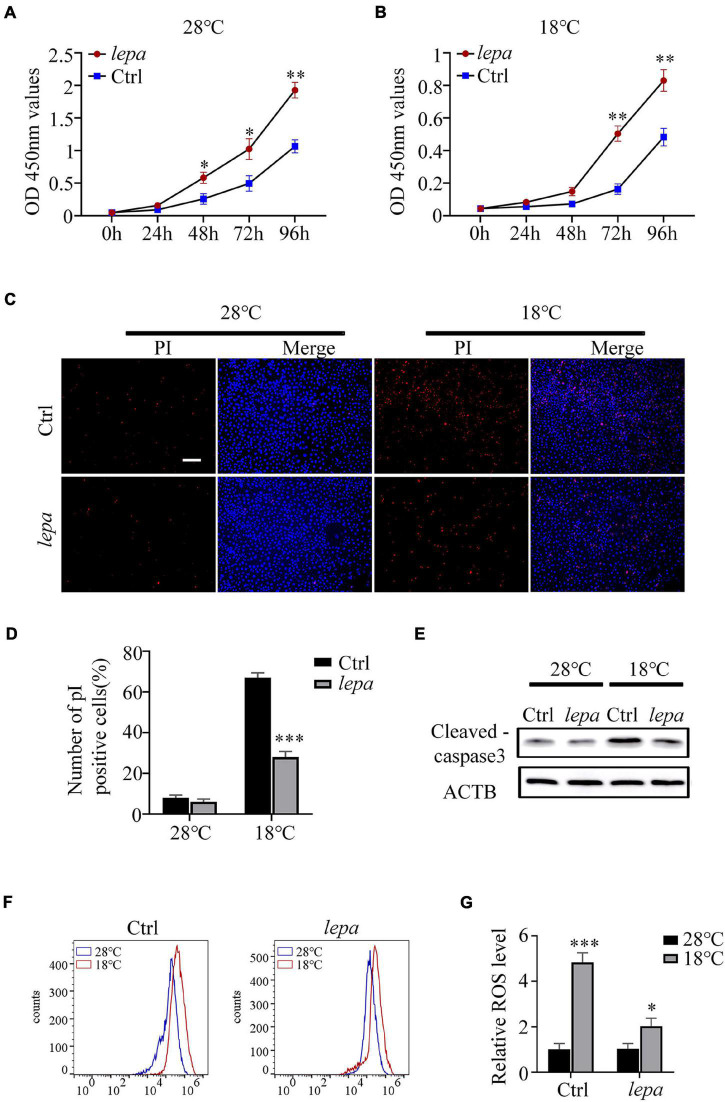
The effect of overexpression of DM-lepa on cells. **(A,B)** Cell proliferation was determined by assay of CCK8. **(C)** Apoptosis was evaluated with Hoechst/PI staining. PI stains dead cells or late-stage apoptotic cells with a red fluorescence, and Hochest stain the nuclei with a bright-blue fluorescence. **(D)** The percentage of apoptotic cells was determined using ImageJ software. **(E)** Protein levels of cleaved-caspase 3 within cells was detected by western blot analysis.β-actin as the loading control. **(F)** ROS was detected by flow cytometry and analyzed using FlowJo software. **(G)** The relative expression of ROS Data were analyzed and graphed using GraphPad Prism 8. The results are presented as mean ± SD (*n* = 3), *P < 0.05, **P < 0.01, ***P < 0.001.

It has been reported that leptin plays an important role in the regulation of cell proliferation and apoptosis (McGaffin et al., 2009; Lam et al., 2010). Accordingly, we determined the cell proliferation and apoptotic index of the DM-*lepa* over-expression group under normal culture temperature (28°C) and cold conditions (18°C). By using CCK-8 assay, we observed that over-expression of DM-*lepa* significantly increased cell proliferation at either normal or cold temperatures (Figures 5A,B). By contrast, the control cells proliferated relatively slowly (Figures 5A,B). No significant difference in apoptotic signals was detected between the control and DM-*lepa* cell groups at 28°C (Figure 5C). However, after cold treatment at 18°C for 5 days, a considerable proportion of apoptotic cells was observed in the control, but a significantly lower proportion of apoptosis was detected in DM-LEPA cells (Figure 5C). Approximately 28% of cells exhibited apoptosis in the DM-*lepa* over-expression group, compared with 67% in the control (Figure 5D). Meanwhile, the key protein levels of caspase-3 in DM-*lepa* over-expression cells were also lower than in the control group during apoptosis under cold temperature, as evaluated by western blotting (Figure 5E). A recent study shows that leptin can modulate ROS generation and thus control cell apoptosis (Blanquer-Rosselló et al., 2015; Hu et al., 2019). Our investigation of ROS generation indicated that over-expression of DM-*lepa* had no effect on the intracellular level of ROS at the normal culture temperature, while significantly decreasing ROS generation under cold treatment (Figures 5F,G); this demonstrates that over-expression of DM-*lepa* decreased apoptosis and stimulated cell proliferation under cold temperature.

### DM-*lepa* Over-Expression Activates the STAT3 Signal Pathway

**FIGURE 6:**
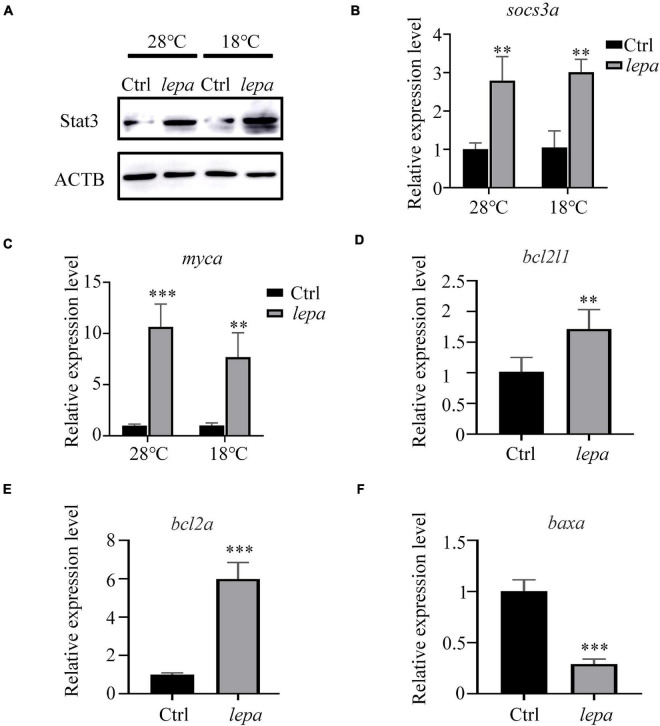
Activation of stat3 signal pathway in DM-lepa-over-expressed cells. **(A)** Western blot analysis of the protein expression level of STAT3 in the cell lysates of the DM-lepa over-expression group and the control group. **(B–F)** A qRT-PCR assay of SOCS3/socs3a, c-myc/myca, Bcl-xl/bcl2l1, Bcl2/bcl2a, and Baxa/baxa mRNAs in control and DM-lepa transfected cells. The results are presented as mean ± SD (*n* = 3), **P < 0.01, ***P < 0.001.

To gain further insight into the role of DM-*lepa* in facilitating proliferation and survival of ZFL cells, we focused on JAK-STAT3 pivotal signal cascades that were reported to regulate proliferation and apoptosis of cells (Takeda et al., 1998; Fernández-Riejos et al., 2008; Mattioli et al., 2009). The immunoblot analysis indicated that over-expression of leptin stimulated STAT3 expression in both normal conditions and under cold stress (Figure 6A). Using transcription of SOCS3/*socs3a* mRNA as a marker of the leptin stimulation involved in the JAK2/STAT3 pathway, the qPCR analysis confirmed significant upregulation of SOCS3/*socs3a* expression in over-expression cells (Figure 6B). We also checked the mRNA levels of related genes, *Bcl-xl*, *Bcl-2*, and *c-myc*, which were reported to be the target genes of STATs when apoptosis was suppressed by the activation of STAT3 (Yu et al., 2009; Abad et al., 2014). The quantified expression levels indicated that over-expression of DM-*lepa* enhanced the expression of c-myc/*myca* both at normal culture temperature and under cold conditions (Figure 6C). The expression of Bcl-xl/*bcl2l1* did not show much difference in normal culture temperature (data not shown), but under cold temperatures it was significantly higher in DM-*lepa* cells than the control (Figure 6D). Bcl2 and Bax are important members of the Bcl2 protein family (Simchi et al., 2020) and Bax was identified as a Bcl2-interacting protein that opposed Bcl2 and promoted cell death (Oltval et al., 1993). Examination of Bcl2/*bcl2a* and Bax/*baxa* transcription showed that over-expression of DM-*lepa* significantly up-regulated the expression of the anti-apoptotic gene Bcl2/*bcl2a* (Figure 6E) while down-regulating Bax/*baxa* (Figure 6F) expression. These data indicate that DM-*lepa* was associated with upregulating anti-apoptotic genes and inhibiting pro-apoptotic genes *via* stimulating the STAT3 signal, thus benefiting cell proliferation and survival.

### DM-*lepa* Over-Expression Decreased p53 Level by Enhancing *mdm2* Expression in Zebrafish Liver Cells Under Cold Temperature

**FIGURE 7:**
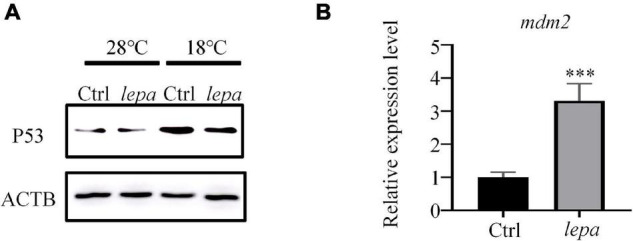
Decreased p53 expression level and enhanced mdm2 expression in ZFL cells under cold temperature. **(A)** Western blot analysis of the protein expression level of P53 in the cell lysates of the DM-lepa over-expression group and the control group cells. **(B)** A qRT-PCR assay of MDM-2/mdm2 in control and DM-lepa transfected cells. The results are presented as mean ± SD (*n* = 3), ***P < 0.001.

To confirm the p53 signaling pathway involved in apoptosis, a p53-specific antibody was used to evaluate the levels of the corresponding protein in over-expression group cells. As shown in Figure 7A, over-expression of DM-*lepa* in ZFL cells decreased p53 levels under cold stress, which was probably negatively regulated by MDM-2, an E3 ubiquitin ligase, *via* a negative feedback loop that is essential to determining cell survival (Wade et al., 2013). Due to the lack of specific antibodies for zebrafish MDM-2, we checked the mRNA expression levels of MDM-2/*mdm2*. As seen in Figure 7B, compared with the control group, over-expression of DM-*lepa* significantly increased the MDM-2/*mdm2* mRNA level under cold conditions. These results accorded with leptin suppressing the activity of p53 and inhibiting p53-mediated apoptosis by upregulating MDM-2/*mdm2* expression under cold temperature.

## Discussion

Leptin is the primary adipogenesis inhibitor produced by mammalian adipocytes (Friedman and Halaas, 1998). It acts on the central nervous system in the brain to regulate energy intake and consumption (Barb, 1999). In addition to regulating fat mass, mammalian leptin also plays a role in glucose homeostasis regulation (Morton, 2007) and reproduction (González et al., 2000). As opposed to mammalian leptin, data on the function of ichthyic leptins are so far preliminary. In this study, we cloned and identified the *leptin-a* gene from *D. mawsoni*, a large slow-growing fish inhabiting the deep ocean at 300–2,500 m off the Antarctic coast where the temperature is perennially sub-zero. The amino acid sequence similarity between the DM-*lepa* mature peptide and *lepa* in other teleosts is relatively low, with only an 18.87% similarity between the DM-*lepa* mature peptide and zebrafish *lepa*, in accord with a previous study reporting that the primary structure of teleost leptin is not conserved. However, DM-*lepa* was predicted to contain four conserved antiparallel α-helices, forming a 4-helix structure consistent with mammals and most fish species. The two conserved disulfide bonds are potentially important in maintaining leptin structure and function. Mutagenic studies have reported that the disulfide bond formed by the two Cys residues is essential for leptin secretion (Keren et al., 2004). In teleosts, leptin homologs are expressed in many tissues, including the liver. For example, *leptin-a* expression is found to be highest in the liver in many fish species, such as *T. rubripes* (Kurokawa et al., 2005), *O. mykiss* (Murashita et al., 2008), *D. rerio* (Gorissen et al., 2009), and *O. latipes* (Kurokawa and Murashita, 2009). By contrast, *lepa* in *D. mawsoni* was highest in muscle. This might relate to the extensive lipid deposits under the skin and in the musculature of *D. mawsoni* (Chen et al., 2019). Similarly in tilapia, a high expression of *lepa* has been reported in the subcutaneous adipose tissue (Liu et al., 2018).

Compared with *lepa* genes of warmer water fishes, the DM-*lepa* gene was subjected to positive selection with 5 positive selection sites (43N, 96K, 103F, 108T, and 142R), three of which (96K, 103F, and 108T) were localized to the 85–119 fragment; this fragment underlies the functional differences between human and non-hominoid leptins (Grasso et al., 1997, 1999; Imagawa et al., 1998), which means that the function of DM-*lepa* may be different from the lepa in all other comparative lineages. At the same time, our evolutionary analysis also confirmed the previous hypothesis that DM-*lepa* has undergone adaptive evolution, and this evolution is closely related to the cold temperature environment. Indeed, many lineages of Antarctic fishes have evolved antifreeze proteins that protect them against the threat of inoculative freezing of their hypoosmotic body fluids.

In a previous study from our laboratory, it was found that the over-expression of calmodulin from Antarctic notothenioid fish increases cold tolerance in tobacco (Yang et al., 2013). Another study showed that elevated LINE activity from an Antarctic notothenioid fish *D. mawsoni* increases the number of viable cells in cold temperatures (Chen et al., 2017). In this study, over-expression of DM-*lepa* significantly increased survival and proliferation in ZFL cells, when stimulated by extreme low temperature. This suggests that DM-*lepa* has a new function as a cell protector at cold temperature and significantly broadened our new knowledge of leptin in regulating cell fate.

It has been reported that leptin activates human peripheral blood B-cells and maintains B-cell homeostasis by inhibiting apoptosis, inducing proliferation, and prolonging survival (Lam et al., 2010; Agrawal et al., 2011). In lean mice, the addition of mouse recombinant leptin significantly increased tracheal epithelial cell proliferation (Tsuchiya et al., 1999). Both lean and obese leptin-deficient mice exhibited increased cardiac apoptosis compared with wild-type mice (McGaffin et al., 2009). Our discovery that the fish leptin has beneficial effects of survival and proliferation in the ZFL cell line is consistent with many previous findings in mammals. However, the finding of reduced ROS content in DM-*lepa* transfected cells in cold stress from this study is novel and implies attenuation of cold stress-induced cellular damage; the underlying mechanism is an interesting avenue for further study. Leptin modulates cell function through activation of the Janus kinase (JAK)-STAT system. The activation of STAT1 or 3 by leptin is believed to involve cell proliferation in the liver (Takahashi et al., 1997; Wang et al., 1997). Leptin’s effects on cell apoptosis are likely mediated through STAT3 to increase anti-apoptotic *bcl-2* and *survivin* gene expression and reduces caspase-3 activity (McGaffin et al., 2009). Inhibition of STAT3 signaling in tumor cells increases the apoptotic rate (Morgan and Macdonald, 2019), and loss of active STAT3 has a significant impact on both cervical cancer cell proliferation and survival (Lis et al., 2017). The downstream targets of STAT3 include factors of anti-apoptotic (*bcl-2*, *bcl-XL*), pro-apoptotic (*baxa*) and proliferative genes (*c-Myc;* Kumar et al., 2016; Baek et al., 2017). Our results clearly show that DM-*lepa* over-expression markedly inhibited Bax/*baxa* expression but increased Bcl-2 expression under cold conditions, eventually resulting in an increased Bcl-2/Bax ratio and cell survival rate.

As the guardian of the genome, p53 is sensitive to environmental factors and is easily activated by a variety of stress signals, especially in response to temperature changes in aquatic organisms (Qian et al., 2020). Li et al. (2018) demonstrated that upregulation of p53 expression in response to low-temperature stress can cause tail malformation of the zebrafish (Li et al., 2018). Qian et al. (2020) reported that genes involved in the p53 signaling pathway were largely affected in the large yellow croaker response to cold stress (Qian et al., 2020). Similarly, p53 mRNA expression was significantly up-regulated in the muscle tissue of *D. rerio* under low-temperature stress (Li et al., 2018). The accumulation of p53 is prevalent in fish responses to cold stress, but over-expression of p53 leads to apoptosis (Chen et al., 1996; Sharp et al., 2014; Armstrong et al., 2017). Over-expression of DM-*lepa* attenuated the expression of p53, suggesting reduced genotoxic stress, corresponding with a lowered ROS level. It has been reported that leptin protects human trophoblasts from serum deprivation-induced cell death by decreased expression of p53 and increased level of MDM-2 (Toro et al., 2014). We hypothesize that the cold-protection effect of DM-*lepa* is beneficial to Antarctic fish cells, which consistently face cold and oxidative stresses. The potential function of the *leptin-a* genes of other teleosts or other Antarctic fishes in coping with cold stress warrants further investigation.

## Data Availability Statement

The original contributions presented in the study are included in the article/Supplementary Material; further inquiries can be directed to the corresponding author/s.

## Ethics Statement

The animal study was reviewed and approved by Shanghai Ocean University. Written informed consent was obtained from the owners for the participation of their animals in this study.

## Author Contributions

YW was responsible for the experimental design and completed the data analysis and wrote the manuscript. HW and LH performed the experiment. LC helped perform the analysis with constructive discussions. All authors contributed to finalizing and approving the manuscript.

## Conflict of Interest

The authors declare that the research was conducted in the absence of any commercial or financial relationships that could be construed as a potential conflict of interest.

## Publisher’s Note

All claims expressed in this article are solely those of the authors and do not necessarily represent those of their affiliated organizations, or those of the publisher, the editors and the reviewers. Any product that may be evaluated in this article, or claim that may be made by its manufacturer, is not guaranteed or endorsed by the publisher.

## Footnotes

1
https://www.ncbi.nlm.nih.gov/orffinder/

2
http://alternate.meme-suite.org/tools/meme

3
https://web.expasy.org/protparam/

4
http://www.cbs.dtu.dk/services/SignalP/

5
http://www.cbs.dtu.dk/services/TMHMM

6
http://www.cbs.dtu.dk/Services/NetPhos

7
http://www.cbs.dtu.dk/services/NetNGlyc

8
https://swissmodel.expasy.org/interactive

## Supplementary Material

Supplementary Table 1 Primers for genes involved in signal transduction pathway.
