# Supplementary material for: Leptin Gene Protects Against Cold Stress in Antarctic Toothfish
Source: Front Physiol. 2021 Dec 15;12:740806. doi: 10.3389/fphys.2021.740806 (PMC8715755; doi:10.3389/fphys.2021.740806)
Supplement: Supplementary Table 1 — Primers for genes involved in signal transduction pathway. [file Table_1.doc]

**Table S1 |** Primers for genes involved in signal transduction pathway.

| Gene name | Forward Primer | Reverse Primer |
| --- | --- | --- |
| *socs3a* | CACTAACTTCTCTAAAGCAGGG | GGTCTTGAAGTGGTAAAACG |
| *myca* | GGCATTCGTTAAACACAAGTGTCA | TCCTCATCGTCGTTGTCGAAG |
| *bcl2l1* | GCGTCGAGTGTGTGGAGAAG | CTCTGCAAAGCGTTCCCATC |
| *bcl2a* | AAATGGAGGTTGGGATGCCT | AAAAGGCTCCGATGGTCACT |
| *baxa* | GTCTTCATCAGAGTGGCCCG | ACCCTGGTTGAAATAGCCTTG |
| *mdm2* | GATCGAGGACCCGGGGATA | GCTGTCCGACTTATGCCTCT |
| *actin* | GATCTGGCATCACACCTTCTAC | TCTTCTCCCTGTTGGCTTTG |
